# 1187. Neurodevelopmental Outcomes of Children with Congenital Cytomegalovirus (cCMV) Infection: Does Antiviral Treatment Matter?

**DOI:** 10.1093/ofid/ofab466.1379

**Published:** 2021-12-04

**Authors:** Margaret R Jia, Alexandra K Medoro, Traci Pifer, Manish Rijal, Teresa Borghese, Ursula M Findlen, S Malhotra, Oliver Adunka, Masako Shimamura, Asuncion Mejias, Nathalie Maitre, Pablo J Sanchez

**Affiliations:** 1 The Ohio State University College of Medicine, Columbus, Ohio; 2 Nationwide Children’s Hospital - The Ohio State University, Columbus, Ohio; 3 Nationwide Children’s Hospital, Columbus, Ohio; 4 The Ohio State University Wexner Medical Center, Columbus, Ohio

## Abstract

**Background:**

cCMV infection is a major contributor to childhood neurologic and cognitive disabilities including sensorineural hearing loss (SNHL). Neonatal treatment with ganciclovir/valganciclovir improves hearing outcomes, but its impact on neurodevelopmental outcomes remains an important knowledge gap. We describe the neurodevelopmental outcomes of children with cCMV infection and evaluate the effect of neonatal antiviral therapy on outcomes.

**Methods:**

Since 2013, infants with cCMV infection referred to Nationwide Children’s Hospital’s NEO-ID Clinic have had a complete evaluation at diagnosis as well as follow-up neurodevelopmental assessments. Pertinent demographic, clinical, laboratory, radiographic, and follow-up data were obtained and managed using REDCap. Neurodevelopmental assessments were performed using Bayley Scales of Infant and Toddler Development (BSID) III/IV (cognitive, language, motor domains) at ~ 24 months of age. The Gross Motor Function Classification System was used to classify functional motor impairment. Neurodevelopmental outcomes were compared by receipt of antiviral therapy in early infancy.

**Results:**

95 infants (mean ± SD; gestational age 35 ± 5 wk, birth weight 2121 ± 948 g; Table 1) with cCMV infection had follow-up neurodevelopmental assessments. 62% had central nervous system involvement, 37% had SNHL, 23% developed cerebral palsy (CP), and 6% were diagnosed with autism spectrum disorder. The majority had normal BSID scores (≥ 85) in cognitive and motor domains (65% and 54%, respectively) while 48% had normal scores in the language domain. 35% had severe impairment (< 70) in ≥ 1 domain (Table 2). 9 children had clinically inapparent cCMV infection; 2 (22%) had abnormalities on BSID testing (1, cognitive score: 80; 1, cognitive, language, and motor scores: 65, 68, 73, respectively). 11 (12%) children, including 6 who received antiviral therapy, had severe neurodevelopmental impairment, with CP and severe (< 70) BSID scores in both the cognitive and motor domains.

Table 1. Demographic and Clinical Characteristics of 95 Children with Congenital CMV Infection by Receipt of Antiviral Treatment

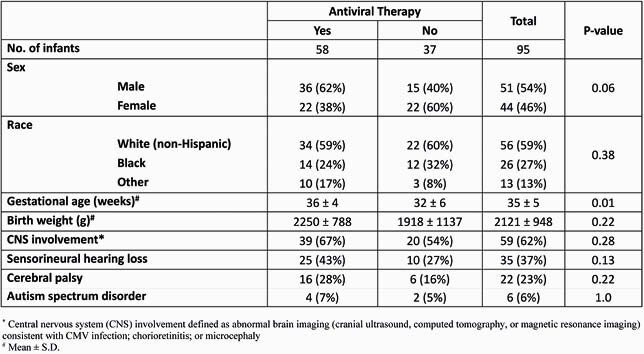

Table 2. Neurodevelopmental Outcomes Based on Testing with the Bayley Scales of Infant and Toddler Development (BSID) III/IV

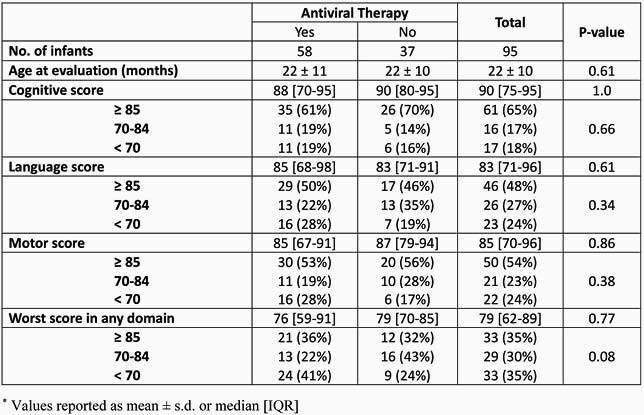

**Conclusion:**

A substantial proportion of children with cCMV infection had moderate (29%) or severe (33%) neurodevelopmental impairment, CP, or autism spectrum disorder, irrespective of antiviral treatment. Urgency exists for antenatal preventive strategies and vaccine development.

**Disclosures:**

**Asuncion Mejias, MD, PhD, MsCS**, **Janssen** (Grant/Research Support, Advisor or Review Panel member)**Merck** (Grant/Research Support, Advisor or Review Panel member)**Roche** (Advisor or Review Panel member)**Sanofi** (Advisor or Review Panel member)

